# *In vitro *and *in vivo *safety evaluation of *Dipteryx alata *Vogel extract

**DOI:** 10.1186/1472-6882-12-9

**Published:** 2012-02-03

**Authors:** Natália Mencacci Esteves-Pedro, Thaisa Borim, Virginia Sbrugnera Nazato, Magali Glauzer Silva, Patricia Santos Lopes, Márcio Galdino dos Santos, Cháriston André Dal Belo, Cássia Regina Primila Cardoso, Eliana Aparecida Varanda, Francisco Carlos Groppo, Marli Gerenutti, Yoko Oshima-Franco

**Affiliations:** 1 School of Pharmacy, University of Sorocaba, UNISO, Rodovia Raposo Tavares km 92.5, Zip code: 18023-000 Sorocaba, SP, Brazil; 2 Federal University of São Paulo, UNIFESP, R. Prof. Artur Riedel, 275, Zip code: 09972-270 Diadema, SP, Brazil; 3 Post-Graduation Course in Environmental Sciences, PGCiamb, Federal University of Tocantins, UFT, Av. NS 15 ALC NO 14, 109 Norte, Zip code: 77001-090 Tocantins, Brazil; 4 Federal University of Pampa, UNIPAMPA, Av. Antonio Trilha, 1847, Zip code: 97300-000 São Gabriel, RS, Brazil; 5 UNESP-São Paulo State University, Faculty of Pharmaceutical Sciences of Araraquara,Department of Biological Sciences, Rodovia Araraquara-Jaú, Km 1, 14801-902 Araraquara, São Paulo, Brazil; 6 Piracicaba Dental School - State University of Campinas (UNICAMP), Av. Limeira 901, Zip code: 13414-903 Piracicaba, SP, Brazil

## Abstract

**Background:**

*Dipteryx alata *Vogel popularly known as "baru" is an important commercial leguminous tree species from the Brazilian Cerrado, which possess medicinal properties, besides its fruits consumption by animals and humans. The use of the "naturally occurring plants" as herbal remedies and foods mainly from leaves, seeds, flowers and roots of plants or extracts require precautions before ensuring these are safe and efficacious. The objective of this study was to evaluate the safety of *D. alata *barks extract.

**Methods:**

Vegetal drugs of *D. alata *barks were submitted to quality control assays and further to the safety assays under 1) *in vitro *parameter by *Salmonella *(Ames) mutagenicity, and 2) *in vivo *parameter on the pregnancy of rats.

**Results:**

The extract was non-mutagenic to any of the assessed strains TA97a, TA98, TA100 and TA102 even after metabolic activation (+S9). All *in vivo *parameters (reproductive ability evaluation, physical development of rat offsprings, and neurobehavioral development assays) showed no changes related to control group.

**Conclusion:**

*D. alata *barks extract is neither mutagenic by the Ames test nor toxic in the pregnancy of rats, with no physical-neurobehavioral consequences on the rat offsprings development.

## Background

*Dipteryx alata *Vogel belongs to Leguminosae family, popularly known as "baru" [1]. Its fruits are consumed by cattle and wild animals [2] and as sweetmeat by humans [3]. Its seeds are edible, nutritive and the oil has medicinal properties [1,2,4], whereas other parts from plant are popularly used as anti rheumatic, tonic and menstrual regulator [5].

One in five individuals taking prescriptions also use herbal remedies or nutritional supplements [6], despite the risks for adverse drug reactions resulting from interactions between herbal remedies and prescription pharmaceuticals. In addition, there are some herbs that are known as teratogens that should under no circumstances be taken during pregnancy, such as *Semen crotonis, Semen pharbitidis, Radix euphorbiae, Radix phytolaccae, Rhizoma sparganii*, and *Rhizoma zedoariae*; whereas other herbs are recognized as potentially dangerous for fetuses, such as *Semen persicae *and *Radix aconiti *[7]. Recently, a new pharmacological anti snake venom was attributed to *D. alata *[8].

All these properties make *D. alata *very attractive for food consumption and medicinal use, although there is no information concerning its safety. In previous studies [9] the IC50 was determined and the obtained value was used to calculate the LD50. Here, we used *in vitro *(Ames test) and *in vivo *(developmental and reproductive toxicology) assays to evaluate the mutagenic properties of *D. alata *barks extract and its effects on reproduction.

## Methods

### Plant material and extraction

The barks of an adult *Dipteryx alata *Vogel tree were collected in Pedro Afonso (Tocantins, Brazil) in December 2007. *D. alata *specimens were identified by Dr. Roseli B. Torres from "Núcleo de Pesquisa e Desenvolvimento do Jardim Botânico", Institute of Agronomy of Campinas. The voucher specimen was deposited (IAC 50629) at the herbarium of the Institute of Agronomy of Campinas (Brazil). The *D. alata *barks were dried at 37°C over 48 hours and then powdered, ground in a mill, macerated (200 g, during 5 days) in 2 L of 70% ethanol and the suspension was then percolated (under protection against light) at 20 drops/min, resulting in a 20% (m/v) hydroalcoholic extract [10]. After, the obtained extract was concentrated under reduced pressure and lyophilized providing 170 g (85% efficiency). It was stored at room temperature without light and humidity until the *in vitro *and *in vivo *toxicological assays were performed.

### Quality control assays of the vegetal drugs

#### Ash and humidity tests

In order to observe their elementary physical and chemical characteristics, *D. alata *powders were submitted to ash and humidity tests [11]. Briefly, 100 g of powder were placed in six calibrated melting pots, which were warmed until total carbonization of the powders was achieved. The melting pots were kept at 650°C and the ashes were then weighed. Results are presented in grams of ashes/100 g of sample. The humidity test was performed by placing 1 g of specimen powder in six calibrated porcelain capsules, which were warmed at 105°C for 4 h and then weighed.

#### Flavonoid content

The content of flavonoids was determined in the hydroalcoholic extract as described elsewhere [12]. The method is based on the UV absorption of Al-Cl_3_-flavonoid complexes and is expressed as total content of quercetin. Briefly, 80% methanol (50 mL) was added to 10 mL of extract and 5 mL of solution were transferred to volumetric flasks and diluted again with 80% methanol (50 mL). Four aliquots (2 mL) of solution were mixed with 2 mL of 5% anhydrous aluminum chloride solution (AlCl_3_; complexing agent) and adjusted to 10 mL with 80% methanol. After 15 min, the absorbance of each sample was read at 420 nm, considering a blank sample containing 80% methanol (8 mL) and 5% AlCl_3 _(2 mL). The percentage of flavonoids (%) was calculated from a standard curve of quercetin (0, 4, 8, 12, and 16 μg/mL) prepared in methanol.

#### Polyphenol content

The content of polyphenols in the hydroalcoholic extract of plant was determined as previously described [13]. Five millilitres of extract was poured in a volumetric flask and distilled water was added to 250 mL, after which a 1 mL aliquot was transferred to another volumetric flask and distilled water added to 25 mL (final solution). Aliquots (1 mL) of the final solution received 1 mL of phosphomolybdotungstic reagent and the final volume (10 mL) was adjusted with 15% sodium carbonate solution. After 30 min, the absorbance of sample was read at 720 nm, considering a blank sample containing 15% sodium carbonate solution. The percentage of polyphenols (%) was determined from a standard curve (5, 10, 15, 20, 25, 30, 35, and 40 μg/mL) of pyrogallol (Sigma Chemical Co., St. Louis, MO, USA).

### Biological tests

#### *In vitro *mutagenicity assay

The *Salmonella *mutagenicity assay was performed using the pre-incubation method for 20 min [14] with *S. typhimurium *strains TA100, TA98, TA97a and TA102 kindly provided by Dr. B. Ames, University of California, Berkeley, CA, USA., with and without metabolic activation. The metabolic activation mixture (S9) was freshly prepared before each test using an Aroclor-1254-induced rat-liver fraction purchased (lyophilized) from Moltox (NC, USA). S9 mix contained 4% (by volume) S9 fraction. The *D. alata *extract was dissolved in dimethyl sulfoxide (DMSO, Sigma Chemical Co., St. Louis, MO, USA) in order to obtain the non toxic concentrations (3.6, 7.1, 14.1, and 21.2 mg/plate) for the *S. typhimurium *strains obtained in a preliminary assay (4.7, 9.4, 18.8, 28.2, and 37.6 mg/plate). Toxicity was apparent either as a reduction in the number of His *+ *revertants, or as an alteration in the auxotrophic background (i.e. background lawn).

Each concentration of *D. alata *extract was added to 500 μL of 0.2 M sodium phosphate buffer (pH 7.4) and 100 μL of each bacterial culture. After 20 min of incubation at 37°C, 2 mL of molten top agar (0.6% agar, histidine and biotin 0.5 mM each, and 0.5% NaCl) was added and the mixture was poured on to a plate containing minimal glucose agar (1.5% Bacto-Difco agar and 2% glucose in Vogel-Bonner medium E). The plates were incubated at 37°C for 48 h and the His(+) revertant colonies were manually counted. The influence of metabolic activation was tested by adding 500 μL of 4% S9 mix to the pre-incubation mixture. All experiments were performed in triplicate. The standard mutagens used as positive controls in the experiments without S9 mix were sodium azide for TA100 (1.25 μg/plate), 4-nitro-*o*-phenylenediamine (10 μg/plate) for TA98 and TA97a and mitomycin for TA102 (0.5 μg/plate). In the tests with metabolic activation, 2-anthramine (1.25 μg/plate) was used for all strains. DMSO was considered as the negative control. Statistical analyses were performed with the SALANAL statistical package software, using the Bernstein et al. [15] model. The mutagenic index (MI), defined as the average number of revertants per plate divided by the average number of revertants per plate in the negative control, was calculated for each dose. A sample was considered positive when the mutagenic index was equal to or greater than two for at least one of the tested concentrations, and if it had a reproducible dose-response curve [16].

#### *In vitro *LD50 of barks *D. alata *hydroalcoholic extract

The value of the LD50 (Lethal Dose to kill 50% of animals) essential for the controlled use of animals in tests *in vivo*, was determined from the barks of *D. alata *hydroalcoholic extract where inhibitory concentration 50%, the concentration required for 50% inhibition (IC50 = 0.164 μg mL^-1^), using the formula: log (LD50 [mg mL^-1^]) = 0.372 × log IC50 (μg mL^-1^) + 2.024 [17]. Thus, the value of the LD50 for extract was 705 mg/kg [9], which justifies the use of 0.5 g/kg, chosen for *in vivo *experimental.

#### In vivo Experimental

##### Preparation of the aqueous D. alata extract

Using the lyophilized *D. alata *extract (see Plant material and extraction), an aqueous extract was freshly prepared in distillated/deionizated water before oral administration.

#### Animals

Male and female adult Wistar rats weighing 160 g to 200 g were supplied by Anilab - Animais de Laboratório (Paulínia, São Paulo, Brazil). All animals were maintained in groups (5 rats per cage), previously housed to laboratory conditions during one week before the experiments at 25 ± 3°C on a 12-h light/dark cycle, and had access to food and water *ad libitum *during all the experimental days. This project (protocol number A079/CEP/2007) was approved by the institutional Committee for Ethics in Research of Vale do Paraiba University (UNIVAP), and the experiments were carried out according to the guidelines of the Brazilian College for Animal Experimentation.

#### Reproductive ability evaluation

This reproductive evaluation method was previously described by Gerenutti et al. [18,19]. Briefly, 26 sexually naive rat females were mated with males (five females with two males per cage). Pregnancy was confirmed through the presence of spermatozoids in vaginal-washing rubbing observed by microscopy analysis [20]. The presence of spermatozoids was considered as the first day of pregnancy. Pregnant females were kept in separate cages. Water and food were supplied *ad libitum *during all the experiment and the consumption of both was monitored daily. For reproductive evaluation each group of 7 females received by gavage 0.5 g/kg/day of *D. alata *extract (group 1) and 0.5 mL/kg/day of deionized water (group 2) from days 0 to 22 of pregnancy.

The weight gain of pregnant females was followed up during the pregnancy. For the teratogenic study each group of 6 females received by gavage 0.5 g/kg/day of *D. alata *extract (group 1) and 0.5 mL/kg/day of deionized water (group 2) from days 0 to 21 of pregnancy. Mothers were anesthetized with the inhalatory halothane (Halotano^®^, Cristalia, Brazil), killed and submitted to a rapidly excision of their uterus. The following macroscopics parameters were evaluated in order to observe the reproductive performance of rats [18,21]:

1) placenta weight (grams)

2) fetus weight (grams)

3) pre-implantation loss (%) = corpora lutea number - implantation number corpora lutea number

4) post-implantation loss (%) = implantation number - alive fetus number implantation number

5) offspring vitality (%)

**Figure 1:**
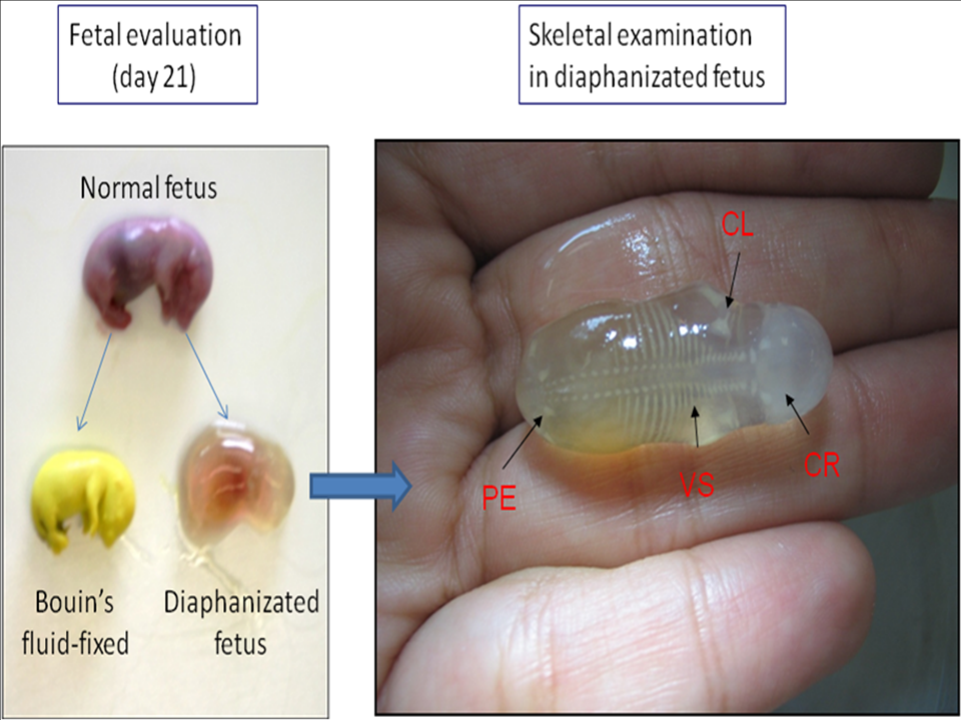
**Gestation evaluation (21st day) under Bouin's fluid-fixed or diaphanizated fetuses**. CL, clavicule. CR, cranio. PE, pelvis. VS, vertebral spine.

The offspring was anesthetized, killed and fixed in Bouin's solution for 24-48 h, replacing it by a 70% hydroalcoholic solution in order to measure the following parameters (in cm): antero-posterior and latero-lateral of cranio; antero-posterior and latero-lateral of thorax; cranio-caudal and tail. Other offspring group was anesthetized, killed, eviscerated and diaphanizated for posterior skeletal examination. Fetuses selected are fixed in ethanol, then "cleared" and stained by a KOH-alizarin red-S technique [22]. Examination includes enumeration of the vertebra, ribs, and other bone structures, degree of ossification, and any fusions or abnormalities in bone shape or position [23]. Figure 1 illustrates fetuses fixed in Bouin's solution or post-diaphanizated.

#### Evaluation of the physical development of rat offspring

The parturition day was defined as first day of life of the litter. On this first day, the offspring was examined externally (macroscopically) and sexed. The same male and female pups were used for the physical and developmental tests. Pups that have been exposed prenatally either to *D. alata *extract or to placebo were submitted to the physical parameters and the behavioral tasks at the same time during the infancy. The following physical development parameters [24] were observed during 21 days: offspring weight; fluff and hair appearing; incisor teeth eruption; ear unsticking/ear opening; eyes opening; testis descent; vagina opening.

#### Neurobehavioral development assays

Two validated methods were adopted for evaluating the neurobehavioral development [19] of pups: latency for uprightness and open field assay (ambulation, rearing frequency, cleaning activity and immobility time). These parameters were measured at 13th day and ended at 21st day.

### Statistical analysis

Data from the different assays were first analyzed regarding distribution and variance homogeneity. Normally distributed data were submitted to comparison between both groups by using Student's *t*-test. Non-normally distributed data were first transformed (log). The Student's *t*-test was also used when the transformation changed the distribution to normal or the Mann-Whitney (Wilcoxon rank sum test) was used if the distribution was still non-normal. The Litchfield and Wilcoxon test [25] were used for evaluation of physical development parameters. The significance level was set at 5%.

## Results and discussion

There is a general belief that herbal remedies are safe because they are natural [26]. Here, it was revealed data such as ashes and humidity - important to attribute a quality control of a given plant. Each 100 g of *D. alata *powder contain 1.55 ± 1.1 g of ashes and 9.5 ± 0.1 g of humidity. It was also expressed the flavonoids and polyphenols content of *D. alata *barks as being 0.42% flavonoids and 3.66% polyphenols, quantified using quercetin calibration curve (Y = 0.0632X + 0.0035, R = 0.998) and pyrogallol calibration curve (Y = 0.1693X - 0.0004, R = 0.999), respectively. Recently, some chemical constituents of *D. alata *were identified [27] such as triterpenoids, isoflavonoids and phenolic acids. These two latter constituents belong to the phenolic compounds, term that embraces a wide range of plant substances, a lot of them of the great biological importance [12].

This is the first time that mutagenicity of *D. alata *extract was investigated using *Salmonella *(Ames test) assay, which has 58% sensitivity to rodent carcinogens, and 73% specificity for noncarcinogens [28]. The *S. typhimurium *used in this assay has a defect in one of genes involved in histidine biosynthesis that renders the cell dependent (auxotrophic) on exogenous histidine. Unless the cell experiences a mutation that revert the dysfunctional gene back to the wild type (prototrophic), the cell becomes disabled when the exogenous histidine is exhausted. For this reason, this assay is referred to as a "reverse" or "back" mutation assay [29], since mutational events can be predictable [30].

Table 1 shows the revertants frequency, the standard deviation and the mutagenicity index (MI) after the treatments with hydroalcoholic extract of *D. alata *bark, in the four different strains of *Salmonella typhimurium*, with or without metabolic activation. The extract was non-mutagenic to any of the assessed strains TA97a, TA98, TA100 and TA102 even after metabolic activation. All values of MI were lower than 2 indicating the absence of any mutagenic activity.

**Table 1:** Mutagenic activity (mean of the number of revertants/plate ± SD) of the bacterial strains TA98, TA100, TA97a and TA102 exposed to *Dipteryx alata *Vogel hydroalcoholic extract, at various concentrations, with (+S9) or without (-S9) metabolic activation

Treatment (mg/plate)	TA98		TA100		*TA97a*		TA102	
	
	- S9	+ S9	- S9	+ S9	- S9	+ S9	- S9	+ S9
0*	25 ± 1.0	23 ± 2.5	155 ± 5.9	207 ± 19.0	197 ± 3.5	188 ± 121	275 ± 14.5	200 ± 9.0

3.6	-	-	-	201 ± 23.5 (0.9)	227 ± 10.8* (1.2)	213 ± 9.9 (1.1)	229 ± 31.8 (0.9)	277 ± 26.9 (1.4)

4.7	25 ± 1.0 (1.0)	23 ± 2.9 (1.0)	135 ± 4.0 (0.9)	-	-	-	-	-

7.1	-	-	-	238 ± 16.5 (1.1)	203 ± 13.5 (1.0)	199 ± 3.5 (1.1)	218 ± 12.1 (0.9)	299 ± 4.6* (1.5)

9.4	40 ± 8.7* (1.6)	24 ± 2.7 (1.0)	149 ± 10.0 (0.9)	-	-	-	-	-

14.1	-	-	-	220 ± 14.8 (1.1)	201 ± 18.9(1.0)	198 ± 12.0 (1.1)	223 ± 3.5 (0.9)	303 ± 5.5* (1.5)

18.8	31 ± 4.4 (1.2)	23 ± 2.0 (1.0)	193 ± 11.7 (1.3)	-	-	-	-	-

21.2	-	-	-	190 ± 16.9 (0.9)	158 ± 31.2 (0.8)	188 ± 7.4 (1.0)	204 ± 15.0 (0.7)	291 ± 1.5 (1.4)

28.2	38 ± 2.9* (1.5)	23 ± 2.1 (1.0)	208 ± 14.0 (1.3)	-	-	-	-	-

37.6	32 ± 6.7 (1.3)	23 ± 2.1 (1.0)	226 ± 40.0* (1.5)	-	-	-	-	-

Control +	2757 ± 208.5^d^	879 ± 46.1^a^	923 ± 134.6^b^	1357 ± 10.6 ^a^	1470 ± 85.9 ^d^	1210 ± 14.5 ^a^	1733 ± 149.7 ^c^	1280 ± 15.3 ^a^

-: not assayed; 0*: negative control (DMSO, 100 μL/plate); Control +: positive control; ^a ^2-Anthramine (1.25 μg/plate); ^b ^Sodium azide (1.25 μg/plate); ^c ^Mitomycin (0.5 μg/plate); ^d^4-nitro-*o*-phenylenediamine (10.0 μg/plate); * *p *< 0.05 (ANOVA). The values in brackets represent the mutagenic index.

Previously, the extract was cytotoxic for TA97a (-S9, +S9), TA100 (+S9) and TA102 (-S9, +S9) at the highest assessed concentrations (28.2 and 37.6 mg/plate). Thus, lower concentrations were used showing the ideal conditions for carrying out a mutagenic assay. These results show a high confidence of *D. alata *as a non-mutagen [28] even under metabolic activation.

**Figure 2:**
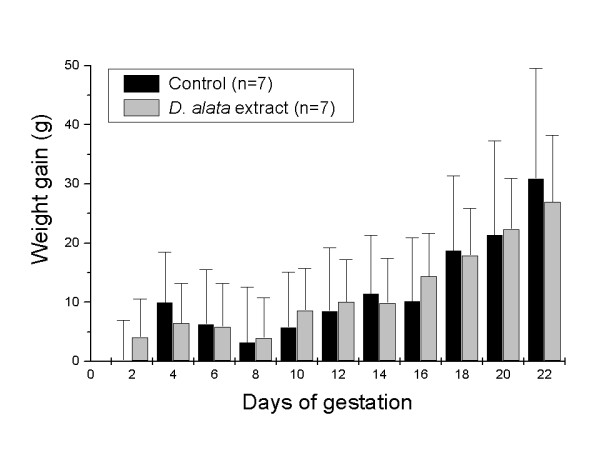
**Effects (mean ± SD) of the *D. alata *aqueous extract on the weight gain of pregnant rats**. There were no statistically significant differences (*p *> 0.05, *t*-test) between the *D. alata *and control groups.

**Figure 3:**
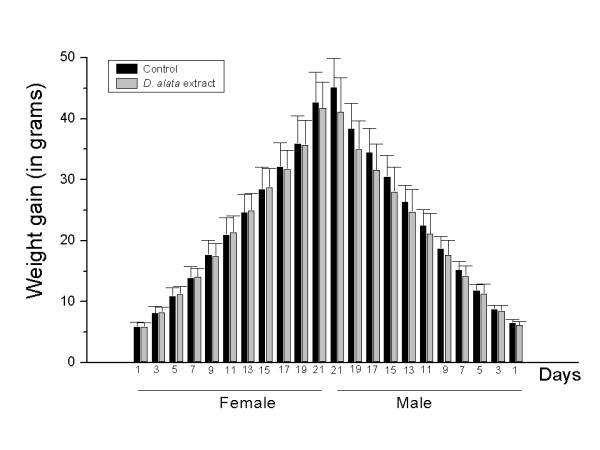
**Weight gain of female (15 animals per group) and male (15 animals per group) pups exposed to *D. alata *extract or deionized water (control) during pregnancy**. There were no statistically significant differences between the groups (*p *> 0.05, *t*-test).

**Figure 4:**
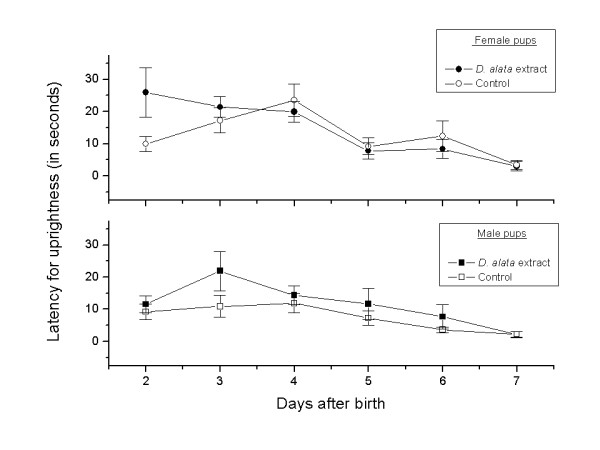
**Latency for uprightness (mean ± SD) of female and male pups exposed to *D. alata *extract and deionized water (control) during the pregnancy**. (*p *> 0.05, *t*-test).

**Figure 5:**
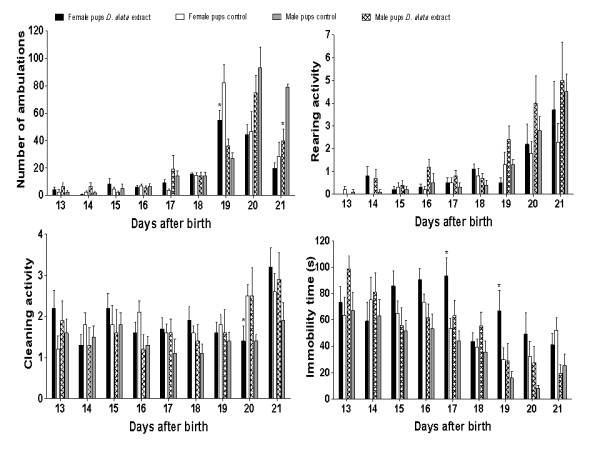
**Open field assay**. Sporadic significant differences in ambulation, cleaning activity and immobility time (* *p *< 0.05, compared to control) were noted.

Additional evidences of *D. alata *safety were provided from *in vivo *assays, since the *D. alata*-tea consumption is a very common habit, mainly during the pregnancy. Here, a set of several parameter was performed, such as the reproductive ability evaluation; weight gain of pregnant females (Figure 2); reproductive performance of pregnant rats (Tables 2 and 3); and offspring survival effects - pups physical development (Figure 3, Table 4); and pups neurobehavioral development (Figures 4 and 5).

Figure 2 shows the mean (± SD) weight gain during the gestation considering water ingestion and food consumption *ad libitum*. At the 0.05 level (*t*-test), the two means (control compared to *D. alata *extract) are not significantly different.

Table 2 shows the reproductive performance of pregnant rats exposed to *D. alata *extract (n = 7) and compared to control group (n = 7). Five parameters were taken into account: 1) placenta weight, 2) pups weight, 3) pre-implantation loss, 4) post-implantation loss; and 5) offspring vitality. The results show that even administrating daily a concentration of 0.5 g/kg no effects were observed on the offsprings (*t*-test, *p *> 0.05).

**Table 2:** Reproductive performance of pregnant rats exposed to *D.alata *Vogel

Parameters	Treatments	
	**Control (n = 7)**	***D. alata *Vogel (0.5 g/kg) (n = 7)**

Placenta weight (in grams)	0.53 ± 0.12	0.49 ± 0.11

Fetus weight (in grams)	2.24 ± 0.55	2.05 ± 0.51

Pre-implantation loss (%)	3.57	4.59

Post-implantation loss (%)	4.9	9.9

Offspring vitality (%)	100	100

Table 3 shows the results obtained after the measurement of offspring external morphological parameters using Bouin's fluid-fixed specimens. No incidence of malformations was seen in this study. There were no statistically significant differences between the animals treated with *D. alata *(0.5 g/kg) or control groups (*p *> 0.05, *t*-test) during pre-birth period.

**Table 3:** Effects of *D.alata *extract administered during rat pregnancy on the offsprings

	Treatment	
**Fetus measurement** **(in cm)**	**Control** **(mean ± SD)** **(n = 39)**	***D. alata *extract (0.5 g/kg)** **(mean ± SD)** **(n = 41)**

Antero-posterior of cranio	11. 74 ± 2. 19	13.69 ± 1.10

Latero-lateral of cranio	7.00 ± 2. 27	8.28 ± 0.72

Antero-posterior of thorax	8.23 ± 1.96	9.28 ± 1. 13

Latero-lateral of thorax	7.94 ± 2. 16	9.89 ± 1.23

Cranio-caudal	26.24 ± 3.07	26.51 ± 3.00

Tail	9.69 ± 1.70	10.56 ± 1.71

No effect on bone tissue was observed during pre-birth period in the offspring exposed to 0.5 g/kg *D. alata *and diaphanizated when compared to the control group (data not shown). The skeletal development (Figure 1), with ossification of clavicule (CL), cranio (CR), pelvis (PE) and vertebral spine (VS) and other structures were normal in both groups with no statistically significant difference between them.

It is well known that when retardation in the uterus growth occurs, a delay or reduction in skeletal ossification and, in some cases, increases in minor skeletal variations is often present. This is observed with test agents that exert their effects through maternal toxicity [23]. Agents known to interfere directly with fetal nutrition, growth factors, and other developmental processes may also induce growth retardation by a direct effect on the fetus [31]. Under some circumstances, the induction of skeletal variations such as supernumerary and wavy ribs is considered the result of maternal toxicity or stress rather than a direct toxic effect on the conceptus [32].

The evaluation of pups' physical development according to offspring weight is shown in Figure 3. Data are presented as mean ± SD weight gain of pups (female and male) previously exposed to aqueous *D. alata *extract during the pregnancy.

After birth, growth can be affected by alterations in maternal care, milk production, or milk ejection reflexes, or even by direct effects on pup's suckling behavior or continued test material exposure through milk [23]. In this study, there were no statistically significant differences between both groups (control and treated), nor between the genders (female and male).

Table 4 shows the result of physical development according to the effective time (in days) necessary to verify the initialization of each parameter. There were no statistically significant difference (*p *> 0.05, Litchfield test) between male and female animals when compared to their respective controls.

**Table 4:** Mean time (in days) necessary to develop of each physical parameter considering male and female pups exposed to aqueous *D.alata *extract or deionized water (control) during pregnancy

Parameters	Male		Female	
	
	Control (n = 15)	*D.alata *extract (n = 15)	Control (n = 15)	*D.alata *extract (n = 15)
Fluff appearing	1-2	1-2	1-3	1-2

Hair appearing	4-5	4-5	3-5	4-5

Incisor teeth eruption	8-11	9-11	8-11	9-10

Ear unsticking/ears opening	12-14	13-15	12-14	13-16

Eyes opening	13-16	13-15	14-16	14-17

Testis descent	20-22	20-24	-	-

Vagina opening	-	-	38-39	34-42

These results taken together show a safety profile of *D. alata *consumption during rat's pregnancy. Absorption by fetus depends on the maternal dose, which in turn depends on the physical and chemical properties of the extract. Toxicity can be direct organ-related toxicity, as hepatocellular necrosis [33]; or indirect, as hyperestrogenism, resulted from excessive consumption of phytoestrogens found in many plants [34]. None adverse effect was observed in the rats as well as in theirs offsprings when 0.5 g/kg *D. alata *extract was administered to the pregnant rats.

The herbal medicine governmental regulation has been addressed mainly to chemicals and drugs that affect the nervous system and behavior, particularly narcotic plants due to the addictive risk and other social issues [26]. The definition of neurotoxicity, however, is "any adverse effect on the structure or function of the nervous system related to exposure to a chemical substance" [35]. In this context, the offspring neurobehavioral development was also evaluated for *D. alata*. Latency for uprightness (Figure 4) and open-field (Figure 5) tests were performed for assessing the neurobehavioral and functional integrity of nervous system.

Figure 4 shows the latency for uprightness for both female and male pups. There were no statistically significant difference (*p *> 0.05, *t*-test) considering both genders.

Figure 5 shows the results on the general physical activity including ambulation, rearing frequency, cleaning activity and immobility time of female and male pups during the open field assay.

Both female and male pups exposed to *D. alata *showed a sporadic reduction on the ambulation parameter, showing statistically significant differences at 19th and 21st days, respectively, when compared to their controls. The number of times the animal rears (defined as any time both front paws leave contact with the floor) showed no statistically differences among groups.

Female offspring exposed to *D. alata *showed a reduction in the cleaning behavioral at 20th day. The immobility time of these animals showed an increase at 17th and 19th days. The results showed in Figure 5 may arise some functional effect in the offspring of mothers exposed to the *D. alata *during pregnancy. However, these effects were sporadic.

## Conclusion

In conclusion, the analysis of all results indicated that the administration of 0.5 g/kg *D. alata *extract during pregnancy in rats is safe, causing no abnormalities and when evaluated by *Salmonella *microsome assay the extract was not mutagenic.

## Competing interests

The authors declare that they have no competing interests.

## Authors' contributions

NME-P, TB, and VSN were students which were responsible by the methodology execution. MGS was responsible for plant quality control. PSL was the responsible by citotoxic study. MGS and CADB were responsible for collection of plant samples obtained in Tocantins. CRPC and EAV were responsible for the mutagenicity study collaboration and interpretation. FCG collaborate in the manuscript redaction and statistical analysis. MG was responsible by *in vivo *assays. YOF was the coordinator and design of all study assays. All authors read and approved the final manuscript

## Pre-publication history

The pre-publication history for this paper can be accessed here:

http://www.biomedcentral.com/1472-6882/12/9/prepub
